# The prevalence of barriers to rearing children aged 0–3 years following China’s new three-child policy: a national cross-sectional study

**DOI:** 10.1186/s12889-022-12880-z

**Published:** 2022-03-12

**Authors:** Liangyu Kang, Wenzhan Jing, Jue Liu, Qiuyue Ma, Shikun Zhang, Min Liu

**Affiliations:** 1 Department of Epidemiology and Biostatistics, School of Public Health, Peking University, No.38, Xueyuan Road, Haidian District, Beijing, 100191 China; 2 Chinese Association for Maternal and Child Health Studies, No.14, Zhichun Road, Haidian District, Beijing, 100190 China

**Keywords:** Rearing children aged 0–3 years, Barrier, Three-child policy, Prevalence, Associated factor

## Abstract

**Background:**

To further optimize birth policy, China implemented a new three-child policy to allow per couple to have up to three children on May 31, 2021.

**Methods:**

A national cross-sectional survey was conducted among 18 to 49-year-old Chinese parents who had at least one child in June 2021. We calculated the prevalence of self-reported childrearing barriers and used univariate logistic regression and multivariate logistic regression to analyze associated factors.

**Results:**

94.7% of the respondents self-reported barriers to rearing children aged 0–3 years, and the biggest barrier included high time cost (39.3%), high parenting cost (36.5%) and high education cost (13.5%). Women (aOR 1.49, 95%CI 1.13,1.96) and people with college degree or above (aOR 3.46, 95%CI 2.08, 5.75) were associated with higher prevalence of childrearing barriers, and people who intended to have a second child (aOR 0.58, 95%CI 0.40, 0.83) and people who intended to have a third child (aOR 0.51,95%CI 0.37, 0.71) were less likely to report childrearing barriers. The biggest barrier was more likely to be high time cost for parents one of whom is only child (aOR1.21, 95%CI 1.03, 1.42) and physical factors for parents both of whom are only child (aOR 1.56,95%CI 1.08, 2.26).

**Conclusions:**

The prevalence of barriers to rearing children aged 0–3 years was high among Chinese people of childbearing age who had children. Full consideration should be given to the barriers of people with different sociodemographic characteristics and people with fertility intention, thus making targeted childrearing policies and supporting measures to reduce the burden on people of childbearing age, encourage suitable couples to have a second or third child and then cope with China’s aging population.

**Supplementary Information:**

The online version contains supplementary material available at 10.1186/s12889-022-12880-z.

## Background

The population, economy and social situation of China are facing new changes and challenges. In order to improve the population structure and actively respond to the aging population, China implemented a new three-child policy to allow per couple to have up to three children on May 31, 2021 [1]. With the arrival of the three-child policy, childrearing has become a focused problem, especially for children aged 0–3 years. Chinese government emphasizes the care of children aged 0–3 years and issued childrearing policies including “Guidance on Promoting the Development of Care Services for Infants and Young Children under 3 Years” [2] and “The Decision to Optimize the Family Planning Policy and Promote Long-term Balanced Population Development” [3] to promote the healthy growth and development of young children.

0–3 years is a critical period for children’s physical and mental development [4]. Ensuring children’s development during this period provides a strong foundation for the future. Children’s early development requires nurturing care, and childrearing can have a significant influence on children’s development [5]. Jeong et al. [6] did a meta-analysis using 102 researches by November 5, 2020, and found that parenting interventions for children during the first 3 years of life are effective for improving early child cognitive, language, motor, socioemotional development, and attachment and reduced behavior problems. Zhou et al. [7] implemented a community-based, integrated and nurturing care intervention among 2745 child-caregiver pairs in four poverty-stricken counties, and found that childcare intervention could significantly prevent developmental delay in children under 3 years in rural China.

For rearing children aged 0–3 years, previous studies mostly focused on childrearing attitude, knowledge and quality [8–10]. There are few researches on childrearing barriers in China and lack of national surveys. In 2009, it was reported that 30.6% of Chinese households with children aged 0–3 years found child-rearing to be much more difficult than before [11]. Recently, Zhang et al. [12] conducted a cross-sectional survey with a sample of 2229 parents of children aged 6–35 months and found that 87.5% of Chinese parents reported experiencing childrearing difficulties, and 31.5% of parents reported experiencing major difficulties. They also found that family having financial problems, and father not joining in child-rearing might face high risk to major childrearing difficulties. Several foreign and domestic studies also showed that sociodemographic characteristics and environment such as parent’s education and family income seem to have influence on childrearing challenges and difficulties [11, 13, 14].

In this study, we performed a national cross-sectional survey on the barriers to rearing children aged 0–3 years in China, hoping to provide scientific evidence for making childrearing policies and supporting measures, help reduce childrearing barriers, ensure the early physical and mental development of children and improve the quality of Chinese population.

## Methods

### Study design and study population

A national anonymous cross-sectional survey was conducted online in June 2021 using a random sampling method on the largest online survey platform in China: Wen Juan Xing (Changsha Ranxing Information Technology Co., Ltd., Hunan, China). A sample database covering over 2.6 million respondents was established by this online platform, whose personal information was confirmed to ensure an authentic, diverse and representative sample [15]. A sample size of 4200 people was indicated to be sufficient to estimate the prevalence of 87.5% (as previously reported in China [12]) with 1% margin of error and 95% confidence level using the formula: $n=\frac{Z_{\alpha /2}^2\times p\left(1-p\right)}{d^2}$[16]. The participants completed the questionnaires online by mobile phone.

A total of 5491 potentially eligible respondents were randomly selected and invited to participate in the survey. After quality control and manual check procedures to exclude ineligible, incomplete, and invalid questionnaires, the final sample consisted of 4406 respondents (80.2%) (flowchart presented in Supplemental Fig. 1).

### Data collection

A self-administered questionnaire was designed to collect information from the participants, including 12 questions about sociodemographic characteristics, 4 questions about reproductive status, 5 questions about fertility intentions and one question about childrearing barriers. The primary outcome was the prevalence of barriers to rearing children aged 0–3 years, which was defined as the proportion of respondents who self-reported childrearing barriers. Fertility intention refers to the unwillingness or willingness to have a second or third child. The only-child situation of parents includes parents neither of whom is only child, parents one of whom is only child, and parents both of whom are only child.

Sociodemographic characteristics included gender, ethnicity (Han and minority), age, residence (rural and urban), educational level (Junior high school or below, Senior high school or equivalent, and College or higher), annual household income (< 30,000, 30,000–80,000, 80,000–120,000, > 120,000 Chinses yuan (CNY)), number of children (1, ≥2), province, and occupation (factory worker, farmer, clerk, public servant, employee, and others). According to the economic development level, the provinces and municipalities were divided into 3 regions, including eastern (Beijing, Tianjin, Hebei, Liaoning, Shanghai, Jiangsu, Zhejiang, Fujian, Shandong, Guangdong, and Hainan), central (Shanxi, Jilin, Heilongjiang, Anhui, Jiangxi, Henan, Hubei, and Hunan) and western (Inner Mongolia, Chongqing, Guangxi, Sichuan, Guizhou, Yunnan, Tibet, Shaanxi, Gansu, Qinghai, Ningxia, and Xinjiang) region [17].

Barriers to rearing children aged 0–3 years was investigated with the question “What do you think is the biggest barrier to rearing 0 to 3-year-old children?” (Answer options: high time cost, high childrearing cost, high education cost, physical factors, others, and no barriers). We defined the respondents who chose the first five options as parents with childrearing barriers. For the biggest barrier, “high time cost” referred to the lack of time to raise children. “High childrearing cost” referred to the heavy economic burden of rearing children. “High education cost” referred to the great pressure to satisfy the education of young children. “Physical factor” referred to the factors related to personal health status.

### Statistical analysis

We used proportion to describe categorical variables, and calculated the prevalence of barriers to rearing children aged 0–3 years. Univariate logistic regression was used to estimate the crude odd ratio (cOR) and its 95% confidence interval (CI). After controlling sociodemographic characteristics (gender, ethnicity, age, residence, educational level, annual household income, number of children, region, and occupation), multivariate logistic regression was adopted to analyze the association between fertility intention, only-child situation of parents and childrearing barriers and then calculated the adjusted odd ratio (aOR) and its 95%CI. Moreover, we analyzed subgroups stratified by number of children. Two-sided *p* values < 0.05 were considered statistically significant. All analyses were performed with R version 4.0.5.

### Patient and public involvement

Patients and the public were not involved in the design and conduct of the study.

## Results

### Sociodemographic characteristics and fertility intention among our study population

Of the 4406 Chinese parents included in our study, 57.9% were women, 95.3% were the Han nationality, 65.5% were urban, 70.9% had a college degree or above, 62.1% had an annual household income of over 80,000 CNY, 68.9% had one child, 31.1% had at least two children, 58.1% lived in the eastern region, and 47.3% were employees (Table 1). 53.0% of the respondents were parents neither of whom is only child, 62.6% intended to have a second child, and 14.8% intended to have a third child.

**Table 1:** The sociodemographic characteristics and barriers to rearing children aged 0–3 years among our study population

Sociodemographic characteristics	Number (n)	Proportion (%)	Proportion of people who have a barrier (%(n))	cOR (95%CI)	aOR (95%CI)
**Total**	4406	100	94.7 (4173)		
**Sex**					
Male	1853	42.1	93.6 (1735)	1	1
Female	2553	57.9	95.5 (2438)	1.44 (1.11, 1.88) *	1.49 (1.13, 1.96) *
**Ethnicity**					
Han	4197	95.3	94.8 (3978)	1	1
Other	209	4.7	93.3 (195)	0.77 (0.45, 1.40)	0.91 (0.50, 1.62)
**Age**					
18–24 years	125	2.8	94.4 (118)	1	1
25–29 years	818	18.6	95.5 (781)	1.25 (0.50, 2.71)	0.99 (0.42, 2.30)
30–34 years	1520	34.5	96.0 (1459)	1.42 (0.58, 2.97)	1.19 (0.52, 2.71)
35–39 years	905	20.5	95.2 (862)	1.19 (0.48, 2.54)	1.14 (0.49, 2.66)
40–49 years	1038	23.6	91.8 (953)	0.67 (0.27, 1.37)	0.81 (0.36, 1.83)
**Residence**					
Rural	1518	34.5	93.7 (1422)	1	1
Urban	2888	65.5	95.3 (2751)	1.36 (1.03, 1.77) *	0.79 (0.55, 1.12)
**Educational level**					
Junior high school or below	462	10.5	89.0 (411)	1	1
Senior high school	819	18.6	91.9 (753)	1.42 (0.96, 2.08)	1.36 (0.89, 2.07)
College or higher	3125	70.9	96.3 (3009)	3.22 (2.26, 4.52) *	3.46 (2.08, 5.75) *
**Annual household income (CNY)**					
< 30,000	658	14.9	91.8 (604)	1	1
30,000–80,000	1013	23.0	94.9 (961)	1.65 (1.11, 2.45) *	1.37 (0.91, 2.06)
80,000–120,000	967	21.9	96.6 (934)	2.53 (1.63, 3.98) *	1.42 (0.88, 2.31)
> 120,000	1768	40.1	94.7 (1674)	1.59 (1.12, 2.24) *	0.67 (0.43, 1.03)
**Number of children**					
1	3036	68.9	95.5 (2899)	1	1
≥ 2	1370	31.1	93.0 (1274)	0.63 (0.48, 0.82) *	0.89 (0.66, 1.21)
**Region**					
Eastern	2558	58.1	95.2 (2436)	1	1
Central	923	20.9	94.0 (868)	0.79 (0.57, 1.10)	0.85 (0.61, 1.20)
Western	925	21.0	93.9 (869)	0.78 (0.56, 1.08)	0.89 (0.63, 1.26)
**Occupation**					
Factory worker	349	7.9	94.6 (330)	1	1
Farmer	318	7.2	87.7 (279)	0.41 (0.23, 0.72) *	0.48 (0.26, 0.87) *
Clerk	322	7.3	92.5 (298)	0.71 (0.38, 1.33)	0.58 (0.31, 1.11)
Public servant	1017	23.1	94.1 (957)	0.92 (0.53, 1.53)	0.58 (0.32, 1.04)
Employee	2083	47.3	96.6 (2012)	1.63 (0.95, 2.69)	1.03 (0.58, 1.84)
Other (including student)	317	7.2	93.7 (297)	0.85 (0.44, 1.64)	0.69 (0.36, 1.35)

*cOR* crude odd ratio, *aOR* adjusted odd ratio, *CNY* Chinese yuan, *CI* confidence interval

* indicates significant at *p*-value < 0.05

### Prevalence of barriers to rearing children aged 0–3 years

**Fig. 1:**
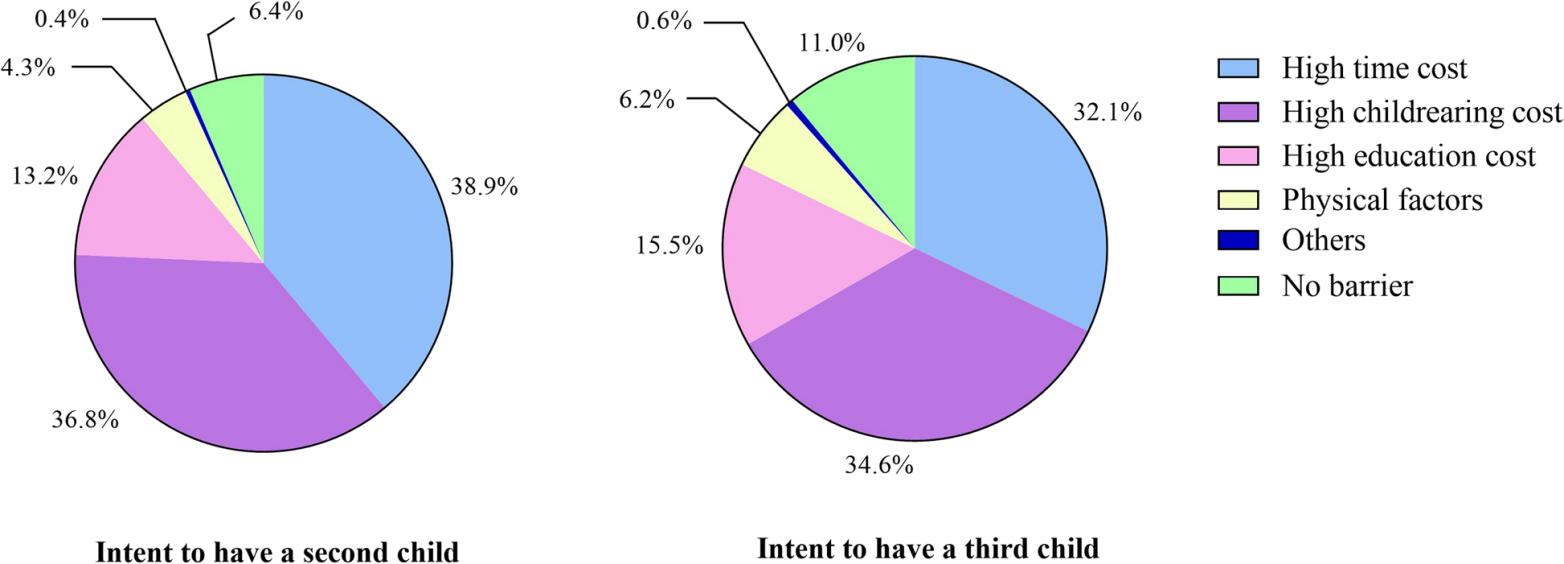
The biggest barrier to rearing children aged 0–3 years among people who intend to have a second/third child

94.7% of the 4406 respondents self-reported barriers to rearing children aged 0–3 years, of which 39.3% reported high time cost, 36.5% reported high childrearing cost, 13.5% reported high education cost, and 5.0% reported physical factors as the biggest barriers (Table 2). High time cost and high childrearing cost were also major barriers among respondents intended to have a second child (75.7%) and those intended to have a third child (66.7%) (Fig. 1).

**Table 2:** The association between the only-child situation of parents, fertility intent and barriers to rearing children aged 0–3 years among our study population

		Having a barrier		High time cost		High childrearing cost		High education cost		Physical factors	
Factor	Number (n (%))	Proportion (%(n))	aOR (95%CI)	Proportion (%(n))	aOR (95%CI)	Proportion (%(n))	aOR (95%CI)	Proportion (%(n))	aOR (95%CI)	Proportion (%(n))	aOR (95%CI)
**Total**	4406	94.7 (4173)		39.3 (1731)		36.5 (1606)		13.5 (593)		5.0 (222)	
**Only-child situation of parents**											
Neither is only child	2337 (53.0)	93.7 (2190)	1	36.7 (858)	1	37.6 (879)	1	13.8 (322)	1	5.0 (118)	1
One parent is only child	987 (22.4)	95.4 (942)	1.07 (0.74, 1.55)	45.3 (447)	1.21 (1.03, 1.42) *	33.0 (326)	0.81 (0.69, 0.96) *	12.7 (125)	1.03 (0.82, 1.30)	4.2 (41)	0.98 (0.67, 1.44)
Both are only child	1082 (24.6)	96.2 (1041)	1.14 (0.76, 1.72)	39.4 (426)	0.85 (0.72, 1.01)	37.1 (401)	1.01 (0.85, 1.20)	13.5 (146)	1.18 (0.92, 1.50)	5.8 (63)	1.56 (1.08, 2.26) *
**Intent to have a second child**											
No	1539 (34.9)	96.8 (1490)	1	40.1 (617)	1	35.9 (552)	1	14.0 (215)	1	6.4 (98)	1
Yes	2867 (65.1)	93.6 (2683)	0.58 (0.40, 0.83) *	38.9 (1114)	1.21 (1.04, 1.42) *	36.8 (1054)	0.93 (0.79, 1.09)	13.2 (378)	0.83 (0.66, 1.04)	4.3 (124)	0.61 (0.42, 0.87) *
**Intent to have a third child**											
No	3742 (84.9)	95.7 (3582)	1	40.6 (1518)	1	36.8 (1376)	1	13.1 (490)	1	4.8 (181)	1
Yes	664 (15.1)	89.0 (591)	0.51 (0.37, 0.71) *	32.1 (213)	0.77 (0.64, 0.93) *	34.6 (230)	0.85 (0.71, 1.02)	15.5 (103)	1.23 (0.95, 1.57)	6.2 (41)	1.59 (1.07, 2.36) *

* indicates significant at *p*-value < 0.05

*aOR* adjusted odd ratio, *CI* confidence interval

The aOR was calculated through the multivariable logistic regression controlling sociodemographic characteristics (sex, ethnicity, age, residence, educational level, annual household income, number of children, region and occupation)

### Related sociodemographic factors of childrearing barriers

Women (aOR 1.49, 95%CI 1.13, 1.96) and people with college degree or above (aOR 3.46, 95%CI 2.08, 5.75) were associated with higher prevalence of childrearing barriers, whereas farmers (aOR 0.48, 95%CI 0.26, 0.87) were associated with lower prevalence (Table 1). Women (aOR 1.17, 95%CI 1.03, 1.33), and people having an annual household income of over 80,000 CNY (aOR 1.50–2.08, all *P* < 0.05) were more likely to report high time cost as the biggest barrier. People having at least 2 children (aOR 1.16, 95%CI 1.00, 1.35) tended to report high childrearing cost, while people with an annual household income of over 120,000 CNY (aOR 0.60, 95%CI 0.48, 0.75) did not. Women (aOR 1.22, 95%CI 1.02, 1.47) and people aged 40–49 years (aOR 1.96, 95%CI 1.02, 3.77) reported high education cost more often, while people having an annual household income of over 80,000 CNY (aOR 0.52–0.63, *P* < 0.05) reported less often (Supplemental Table 1, Supplemental Fig. 2).

### The association between fertility intention, only-child situation of parents and childrearing barriers

Multivariate logistic regression models showed that people who intended to have a second child (aOR 0.58, 95%CI 0.40, 0.83) and people who intended to have a third child (aOR 0.51,95%CI 0.37, 0.71) were associated with less childrearing barriers (Table 2). People who intended to have a second child (aOR 1.21, 95%CI 1.04, 1.42) and parents one of whom is only child (aOR1.21, 95%CI 1.03, 1.42) were more likely to report high time cost as the biggest barrier, while people who intended to have a third child (aOR 0.77, 95%CI 0.64, 0.93) were less likely to report (Table 2). Parents one of whom is only child (aOR 0.81, 95%CI 0.69, 0.96) were related to less reported high childrearing cost. People who intended to have a third child (aOR 1.59, 95%CI 1.07, 2.36) and parents both of whom are only child (aOR 1.56, 95%CI 1.08, 2.26) were more likely to report physical factors, while people who intended to have a second child (aOR 0.61, 95%CI 0.42, 0.87) were less likely to report. In subgroup analysis, the association between fertility intention and childrearing barriers were stable (Table 3).

**Table 3:** The barriers to rearing children aged 0–3 years among our study population stratified by number of children

	Number of children = 1			Number of children ≥ 2		
Factor	Number (n (%))	Proportion of people who have a barrier (% (n))	aOR (95%CI)	Number (n (%))	Proportion of people who have a barrier (% (n))	aOR (95%CI)
**Total**	3036	95.5 (2899)		1370	93.0 (1274)	
**Sex**						
Male	1249 (41.1)	94.5 (1180)	1	606 (44.1)	91.9 (555)	1
Female	1787 (58.9)	96.2 (1719)	1.27 (0.89, 1.82)	766 (55.9)	93.9 (719)	1.57 (0.99, 2.47)
**Residence**						
Rural	813 (26.8)	94.6 (769)	1	705 (51.5)	92.6 (653)	1
Urban	2223 (73.2)	95.8 (2130)	0.91 (0.57, 1.46)	665 (48.5)	93.4 (621)	0.66 (0.38, 1.15)
**Educational level**						
Junior high school or below	150 (4.9)	88.7 (133)	1	312 (22.8)	89.1 (278)	1
Senior high school	448 (14.8)	91.7 (411)	1.19 (0.61, 2.33)	371 (27.1)	92.2 (342)	1.42 (0.80, 2.51)
College or higher	2438 (80.3)	96.6 (2355)	3.14 (1.50, 6.56) *	687 (50.1)	95.2 (654)	3.31 (1.56, 7.02) *
**Annual household income (CNY)**						
< 30,000	336 (11.1)	95.9 (312)	1	322 (23.5)	90.7 (292)	1
30,000–80,000	589 (19.4)	95.3 (561)	1.33 (0.73, 2.42)	424 (30.9)	94.3 (400)	1.46 (0.82, 2.61)
80,000–120,000	706 (23.3)	96.9 (684)	1.41 (0.74, 2.72)	261 (19.1)	95.8 (250)	1.52 (0.70, 3.28)
> 120,000	1405 (46.3)	95.5 (1342)	0.71 (0.39, 1.29)	363 (26.5)	91.5 (332)	0.57 (0.29, 1.11)
**Occupation**						
Factory worker	181 (6.0)	92.3 (167)	1	168 (12.3)	97.0 (163)	1
Farmer	98 (3.2)	86.7 (85)	0.71 (0.30, 1.69)	220 (16.1)	88.2 (194)	0.21 (0.08, 0.59) *
Clerk	202 (6.7)	94.6 (191)	1.27 (0.54, 2.99)	120 (16.1)	89.2 (107)	0.21 (0.07, 0.63) *
Public servant	733 (24.1)	94.7 (694)	1.15 (0.56, 2.36)	284 (20.7)	92.6 (263)	0.25 (0.09, 0.74) *
Employee	1657 (54.6)	96.7 (1602)	1.63 (0.81, 3.28)	426 (31.1)	96.2 (410)	0.54 (0.18, 1.62)
Other (including student)	165 (5.4)	97.0 (160)	2.59 (0.87, 7.72)	152 (11.1)	90.1 (137)	0.21 (0.07, 0.62) *
**Intent to have a second child**						
No	1539 (50.7)	96.8 (1490)	1	0	–	–
Yes	1497 (49.3)	94.1 (1409)	0.60 (0.39, 0.92) *	100 (1274)	–	–
**Intent to have a third child**						
No	2696 (88.8)	96.3 (2597)	1	1046 (76.4)	985 (94.2)	1
Yes	340 (11.2)	88.8 (302)	0.41 (0.26, 0.65) *	324 (23.6)	289 (89.2)	0.61 (0.39, 0.97) *

*aOR* adjusted odd ratio, *CI* confidence interval, *CNY* Chinese yuan

The aOR was calculated through the multivariable logistic regression controlling variables including ethnicity, age, region and the only-child situation of parents

* indicates significant at *p*-value < 0.05

## Discussion

In this study, we conducted a national represented cross-sectional study in 2021, right after the new three-child policy, to estimate the prevalence of childrearing barriers and analyze related factors, thereby helping make childrearing policies and supporting measures. We found that 94.7% of 4406 Chinese adults aged 18–49 years who had children self-reported barriers to rearing children aged 0–3 years. The biggest barrier included high time cost, high childrearing cost and high education cost. For related factors, women and well-educated people were associated with higher prevalence of barriers, while people who intended to have a second or third child were less likely to report childrearing barriers. Attention should be paid to childrearing barriers among children aged 0–3 years following the change of family planning policy.

The prevalence of childrearing barriers in our study was close to previous studies [11, 12]. Zhao et al. found that 88.2% of Chinese caregivers of children aged below 3 years reporting parenting difficulties in 2010 [11]. Zhang et al. [12] performed a cross-sectional self-reporting survey with a sample of 2229 parents of children aged 6–35 months in 2017 and found that 87.5% of Chinese parents reported experiencing childrearing difficulties. Our result was slightly higher probably due to the different characteristics of the study population. Zhang et al. only investigated 15 cities in China. Moreover, with the development of society and economy, people are increasingly busy with work and do not have enough time to raise children, leading to an increase in the prevalence of childrearing barriers. Additionally, rising prices have added to the economic burden of childrearing [18]. Therefore, our results suggest that it is important to help parents reduce childrearing barriers.

We found that women were more likely to report childrearing barriers than men, consistent with previous researches [19, 20]. Although women traditionally play a significant role in family and childcare, more and more women enter workforce nowadays. Previous studies showed that it is difficult for working women to balance childcare and career because of incomplete supporting system for them in China [21]. Additionally, father involvement in children’s early upbringing is a key source of positive child developmental outcomes [22–24]. However, fathers’ involvement in parenting was less than mothers’ in Chinese families [10]. Therefore, childrearing policies and supporting measures should be improved to help women to juggling work and childcare and encourage fathers to participate in childrearing.

Well-educated people reported childrearing barriers more often. And this was consistent with previous study [13]. The reason might be their more attention to childrearing and education, more investment and busier work. Our results also showed that farmers self-reported barriers less often. This be ascribed to their outdated childrearing concepts, lack of scientific childrearing knowledge, insufficient investment, and lower parenting costs in rural areas [25], making them to feel easy to raise children aged 0–3 years. A survey conducted in 1715 rural households in western China found that the average parenting knowledge score of sample caregivers (0.52) is much lower than the expected average score (0.72) and parental investments are poor in rural areas [8]. Therefore, it is necessary to strengthen education of parenting knowledge and guide farmers to form scientific childrearing concepts.

Notably, parents one of whom is only child were more likely to report high time cost as the biggest barrier, and parents both of whom are only child were more likely to report physical factors. After one-child policy for 36 years and universal two-child policy for only 5 years in China [26], many only-children became parents and might face considerable childrearing barriers. Besides time cost, economic cost, physical factors and other “hard barriers”, only-child couples might face psychological and cultural barriers and need more time to adjust and accept [27]. Moreover, only-child couples tended to have more than one child [28]. Therefore, targeted strategies are needed for only-child parents to support their childrearing.

For the association between fertility intention and childrearing barriers, people who intended to have a second child and people who intended to have a third child were less likely to report barriers. This finding was similar to previous studies in the context of two-child policy [29, 30]. A cross-sectional study of 11,991 Chinese women on fertility intention in 2016 and 2017 indicated that economic, health, childrearing, and educational barriers were associated with a lower intent to have a second child [30]. Conversely, people with fertility intention might have positive attitude towards childrearing. Nevertheless, because of their fertility potential, sustained efforts to reduce their barriers to rearing children aged 0–3 years are required.

For high time cost, our findings showed that people intended to have a second child were more likely to report high time cost as the biggest barrier, while people intended to have a third child were less likely to report. The potential reason is that people with sufficient time would consider having a third child. With the development of society and the popularization of education, Chinese people of childbearing age are widely involved in social production. Busy working parents often leave their children to their grandparents to raise [31, 32], which might mitigate this problem but bring childrearing pressure to grandparents [33]. Moreover, left-behind children need more attention due to the detrimental influence of parental migration and poor rearing environment [34–36]. Therefore, sufficient parental leave, available and qualified childcare services, and other supporting measures should be provided to reduce high time cost of rearing children aged 0–3 years.

For high childrearing cost, our study suggested that 36.5% of Chinese parents of childbearing age reported high childrearing cost as the biggest barrier. Based on the data from China Family Panel Studies(CFPS) in 2013, the average direct consumption expenditure of 0 to 5-year-old children (including food, clothing, shelter, childcare, education and medical care) was 62,726 Chinese yuan [37]. The financial burden of childrearing is also a substantial barrier to fertility intention. Liu et al. [30] found that 47.7% of Chinese women of childbearing age reported economic barrier as the main obstacle to having a second child. In order to reduce economic cost of childrearing, childbirth allowance for parents with a second or third child, and strengthening price regulation of childcare products and services are expected.

For high education cost, we found that 13.5% of Chinese adults aged 18–49 years who had children reported high education cost as the biggest barrier, consistent with the results of previous cross-sectional surveys [30]. Nowadays, Chinese parents attached great importance to early education of children aged 0–3 years [38]. However, the proportion of young children’s enrollment in various childcare institutions is less than 5% in China, far lower than 50% in some developed countries [39]. Additionally, there exist many problems such as uneven quality of childcare services, and shortage of teachers and professionals [40]. Therefore, it is necessary to make relevant policies and measures to encourage the development of childcare and early education institutions and strengthen regulation.

For physical barriers, our results indicated that people who intended to have a third child were more likely to report physical factors as the biggest barrier, while people who intended to have a second child were less likely to report. A cross-sectional study among Japanese mother showed that mothers aged 40 years or older had a high risk of facing difficulties with childrearing [41]. People are younger and healthier when they have a second child, thus downplaying physical factors. In contrast, people intended to have a third child are concerned about their health because they are older when they have a third child. Therefore, following the three-child policy, targeted childrearing policies and measures for older parents are needed.

The ongoing COVID-19 pandemic might bring about difficulties in childrearing [42]. The United Nations Educational, Scientific and Cultural Organization estimates 1.38 billion children are out of school or child care [43]. The economic impact of the pandemic increases the financial burden of rearing children aged 0–3 years [43]. Moreover, the health risks and fear connected to COVID-19 influence parents’ levels of stress and consequently children’s well-being [44]. Therefore, it is essential to make effective strategies to strengthen childrearing, and protect a future for children during the COVID-19 pandemic.

### Strengths and limitations

The main strength of our study is that it is the first to understand the barriers to rearing children aged 0–3 years and the association between fertility intention and childrearing barriers among Chinese parents following the new three-child policy. The estimated prevalence of barriers could provide scientific evidence for the need of making childrearing policies and supporting measures, and the analysis of related factors could help formulate targeted policies and measures for people with different sociodemographic characteristics and fertility intention, thereby reducing childrearing barriers and guaranteeing child health. However, there are some limitations. First, we collected data using online questionnaire, so that people who were not internet users were not included in our study. Nevertheless, there were 989 million internet users in China by December 2020, and 99.7% of them surf the internet by mobile phone [45]. Additionally, internet use was more prevalent in people of childbearing age than in other age groups. Second, our study was cross-sectional and could not demonstrate causal association. Third, because of lacking occupation option like “immigrant workers” in our questionnaire, we could not measure the barriers among immigrant Chinese who face more childrearing difficulties [46]. Last, the coronavirus disease 2019 (COVID-19) pandemic might have an impact on our results [42].

## Conclusions

In conclusion, 94.7% of Chinese people of childbearing age who had children self-reported barriers to rearing children aged 0–3 years. The biggest barrier mainly included high time cost, high childrearing cost and high education cost. The people who intended to have a second child and people who intended to have a third child were less likely to report childrearing barriers. Full consideration should be given to the barriers of people with different sociodemographic characteristics and people with fertility intention, thus making targeted childrearing policies and supporting measures to reduce the burden on people of childbearing age, encourage suitable couples to have a second or third child and then cope with China’s aging population.

## Supplementary Information

**Additional file 1: Supplemental Table 1.** The biggest barrier to rearing children aged 0–3 years among our study population.

**Additional file 2: Supplemental Fig. 1.** Flowchart of the study profile.

**Additional file 3: Supplemental Fig. 2.** The biggest barrier to rearing children aged 0–3 years among our study population with different sociodemographic characteristics. (A) High time cost, (B) High childrearing cost, (C) High educational cost, (D) Physical factors.

## Data Availability

The datasets used and/or analysed during the current study are available from the corresponding author on reasonable request.

## Abbreviations

cOR: crude odd ratio
CI: confidence interval
aOR: adjusted odd ratio
