# Supplementary material for: The prevalence of barriers to rearing children aged 0–3 years following China’s new three-child policy: a national cross-sectional study
Source: BMC Public Health. 2022 Mar 12;22:489. doi: 10.1186/s12889-022-12880-z (PMC8917473; doi:10.1186/s12889-022-12880-z)
Supplement: Supplementary file 1 — Additional file 1: Supplemental Table 1. The biggest barrier to rearing children aged 0–3 years among our study population. [file 12889_2022_12880_MOESM1_ESM.docx]

Supplemental Table 1 The biggest barrier to rearing children aged 0-3 years among our study population

|  | **High time cost** | | **High childrearing cost** | | **High education cost** | | **Physical factors** | |
| --- | --- | --- | --- | --- | --- | --- | --- | --- |
| **Sociodemographic characteristics** | **Proportion**  **(% (n))** | **aOR (95%CI)** | **Proportion**  **(% (n))** | **aOR (95%CI)** | **Proportion**  **(% (n))** | **aOR (95%CI)** | **Proportion**  **(% (n))** | **aOR (95%CI)** |
| **Total** | 39.3 (1731) |  | 36.5 (1606) |  | 13.5 (591) |  | 5.0 (222) |  |
| **Sex** |  |  |  |  |  |  |  |  |
| Male | 37.5 (694) | 1 | 39.1 (725) | 1 | 12.1 (224) | 1 | 4.5 (84) | 1 |
| Female | 40.6 (1037) | 1.17 (1.03, 1.33) * | 34.5 (881) | 0.79 (0.70, 0.90) * | 14.5 (369) | 1.22 (1.02, 1.47) * | 5.4 (138) | 1.33 (0.99, 1.76) |
| **Ethnicity** |  |  |  |  |  |  |  |  |
| Han | 39.9 (1674) | 1 | 36.5 (1531) | 1 | 13.2 (555) | 1 | 4.7 (199) | 1 |
| Other | 27.3 (57) | 0.68 (0.49, 0.94) * | 35.9 (75) | 0.94 (0.70, 1.27) | 18.2 (38) | 1.25 (0.86, 1.82) | 11.0 (23) | 2.38 (1.47, 3.87) * |
| **Age** |  |  |  |  |  |  |  |  |
| 18-24 years | 40.8 (51) | 1 | 40.0 (50) | 1 | 8.8 (11) | 1 | 4.8 (6) | 1 |
| 25-29 years | 41.7 (341) | 0.88 (0.60, 1.31) | 37.4 (306) | 0.94 (0.64, 1.39) | 12.7 (104) | 1.66 (0.86, 3.21) | 3.3 (27) | 0.68 (0.27, 1.70) |
| 30-34 years | 40.9 (621) | 0.86 (0.59, 1.27) | 38.2 (581) | 0.96 (0.66, 1.41) | 12.6 (191) | 1.67 (0.88, 3.18) | 4.1 (62) | 0.86 (0.36, 2.05) |
| 35-39 years | 39.2 (335) | 0.90 (0.60, 1.33) | 37.1 (336) | 0.86 (0.58, 1.27) | 14.1 (128) | 1.86 (0.96, 3.58) | 4.2 (38) | 0.91 (0.37, 2.24) |
| 40-49 years | 35.0 (363) | 0.80 (0.54, 1.19) | 32.1 (333) | 0.69 (0.47, 1.02) | 15.3 (159) | 1.96 (1.02, 3.77) * | 8.6 (89) | 1.90 (0.80, 4.52) |
| **Residence** |  |  |  |  |  |  |  |  |
| Rural | 36.0 (546) | 1 | 39.1 (594) | 1 | 13.8 (210) | 1 | 4.2 (63) | 1 |
| Urban | 41.0 (1185) | 0.89 (0.76, 1.04) | 35.0 (1012) | 0.98 (0.84, 1.15) | 13.3 (383) | 1.10 (0.88, 1.37) | 5.5 (159) | 1.26 (0.87, 1.82) |
| **Educational level** |  |  |  |  |  |  |  |  |
| Junior high school or below | 29.0 (134) | 1 | 40.7 (188) | 1 | 14.3 (66) | 1 | 4.1 (19) | 1 |
| Senior high school | 32.6 (267) | 0.95 (0.73, 1.24) | 36.1 (296) | 0.91 (0.71, 1.17) | 16.2 (133) | 1.33 (0.95, 1.86) | 6.2 (51) | 1.52 (0.86, 2.70) |
| College or higher | 42.6 (1330) | 1.03 (0.78, 1.35) | 35.9 (1122) | 1.11 (0.86, 1.44) | 12.6 (394) | 1.28 (0.89, 1.84) | 4.9 (152) | 1.18 (0.64, 2.20) |
| **Annual household income (RMB)** |  |  |  |  |  |  |  |  |
| < 30 000 | 27.7 (182) | 1 | 40.4 (266) | 1 | 17.9 (118) | 1 | 4.7 (31) | 1 |
| 30 000-80 000 | 31.7 (321) | 1.14 (0.91, 1.42) | 41.0 (415) | 1.01 (0.83, 1.24) | 17.0 (172) | 0.94 (0.72, 1.22) | 4.6 (47) | 1.01 (0.63, 1.64) |
| 80 000-120 000 | 39.3 (380) | 1.50 (1.19, 1.88) * | 39.5 (382) | 0.90 (0.73, 1.12) | 12.1 (117) | 0.63 (0.47, 0.85) * | 5.3 (51) | 1.31 (0.80, 2.15) |
| > 120 000 | 48.0 (848) | 2.08 (1.67, 2.60) * | 30.7 (543) | 0.60 (0.48, 0.75) * | 10.5 (186) | 0.52 (0.39, 0.70) * | 5.3 (93) | 1.36 (0.83, 2.21) |
| **Number of children** |  |  |  |  |  |  |  |  |
| 1 | 42.0 (1274) | 1 | 35.0 (1064) | 1 | 12.9 (391) | 1 | 5.2 (158) | 1 |
| ≥ 2 | 33.4 (457) | 0.86 (0.74, 1.00) | 39.6 (542) | 1.16 (1.00, 1.35) * | 14.7 (202) | 1.02 (0.83, 1.25) | 4.7 (64) | 0.85 (0.61, 1.17) |
| **Region** |  |  |  |  |  |  |  |  |
| Eastern | 39.4 (1007) | 1 | 36.7 (940) | 1 | 13.8 (352) | 1 | 4.9 (125) | 1 |
| Central | 40.7 (376) | 1.28 (1.09, 1.51) * | 36.3 (335) | 0.90 (0.77, 1.06) | 11.5 (106) | 0.70 (0.55, 0.89) * | 5.0 (46) | 0.95 (0.66, 1.36) |
| Western | 37.6 (348) | 1.18 (1.00, 1.39) * | 35.8 (331) | 0.90 (0.76, 1.06) | 14.6 (135) | 0.89 (0.71, 1.11) | 5.5 (51) | 0.93 (0.65, 1.33) |
| **Occupation** |  |  |  |  |  |  |  |  |
| Factory worker | 30.4 (106) | 1 | 43.6 (152) | 1 | 16.0 (56) | 1 | 4.0 (14) | 1 |
| Farmer | 26.4 (84) | 0.87 (0.61, 1.23) | 37.7 (120) | 0.81 (0.59, 1.12) | 16.0 (51) | 0.97 (0.63, 1.49) | 6.3 (20) | 1.72 (0.82, 3.61) |
| Clerk | 36.3 (117) | 1.08 (0.78, 1.51) | 36.3 (117) | 0.84 (0.61, 1.15) | 14.9 (48) | 0.96 (0.62, 1.47) | 5.0 (16) | 1.17 (0.56, 2.48) |
| Public servant | 38.2 (388) | 1.08 (0.81, 1.44) | 33.8 (344) | 0.77 (0.59, 1.02) | 15.3 (156) | 1.04 (0.72, 1.50) | 6.2 (63) | 1.27 (0.67, 2.42) |
| Employee | 45.0 (937) | 1.31 (1.00, 1.72) | 35.8 (746) | 0.86 (0.66, 1.11) | 11.3 (235) | 0.80 (0.56, 1.13) | 4.4 (91) | 1.03 (0.55, 1.92) |
| Other (including student) | 31.2 (99) | 0.86 (0.61, 1.21) | 40.1 (127) | 0.98 (0.71, 1.35) | 14.8 (47) | 0.94 (0.61, 1.46) | 5.7 (18) | 1.34 (0.64, 2.81) |

* indicates significant at p-value <0.05

aOR: adjusted odd ratio; CI: confidence interval; RMB: renminbi.
