# Supplementary figures and images for: The prevalence of barriers to rearing children aged 0–3 years following China’s new three-child policy: a national cross-sectional study
Source: BMC Public Health. 2022 Mar 12;22:489. doi: 10.1186/s12889-022-12880-z (PMC8917473; doi:10.1186/s12889-022-12880-z)

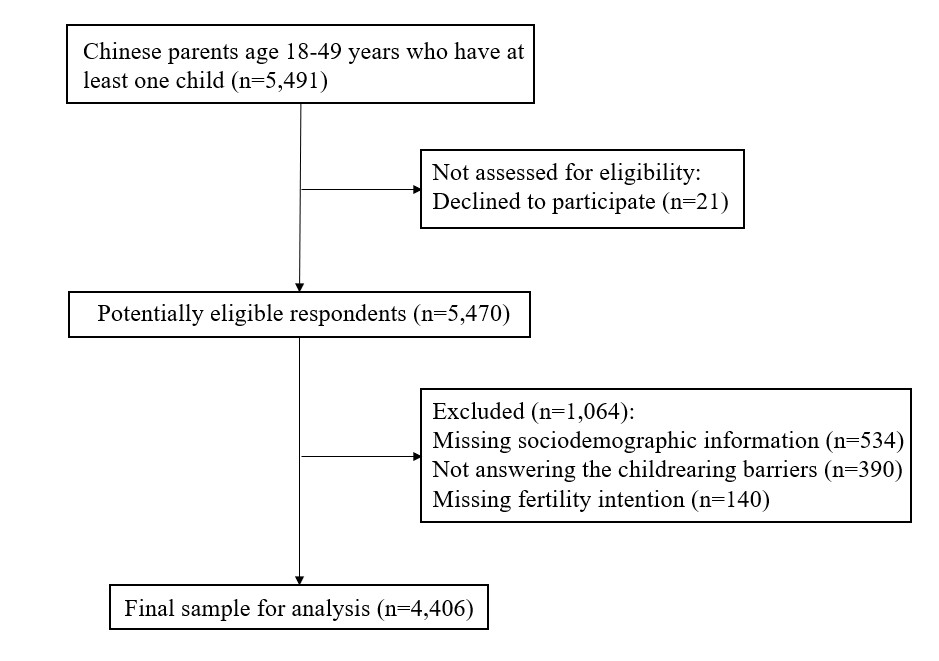

Supplement: Supplementary file 2 — Additional file 2: Supplemental Fig. 1. Flowchart of the study profile. [file 12889_2022_12880_MOESM2_ESM.jpg]

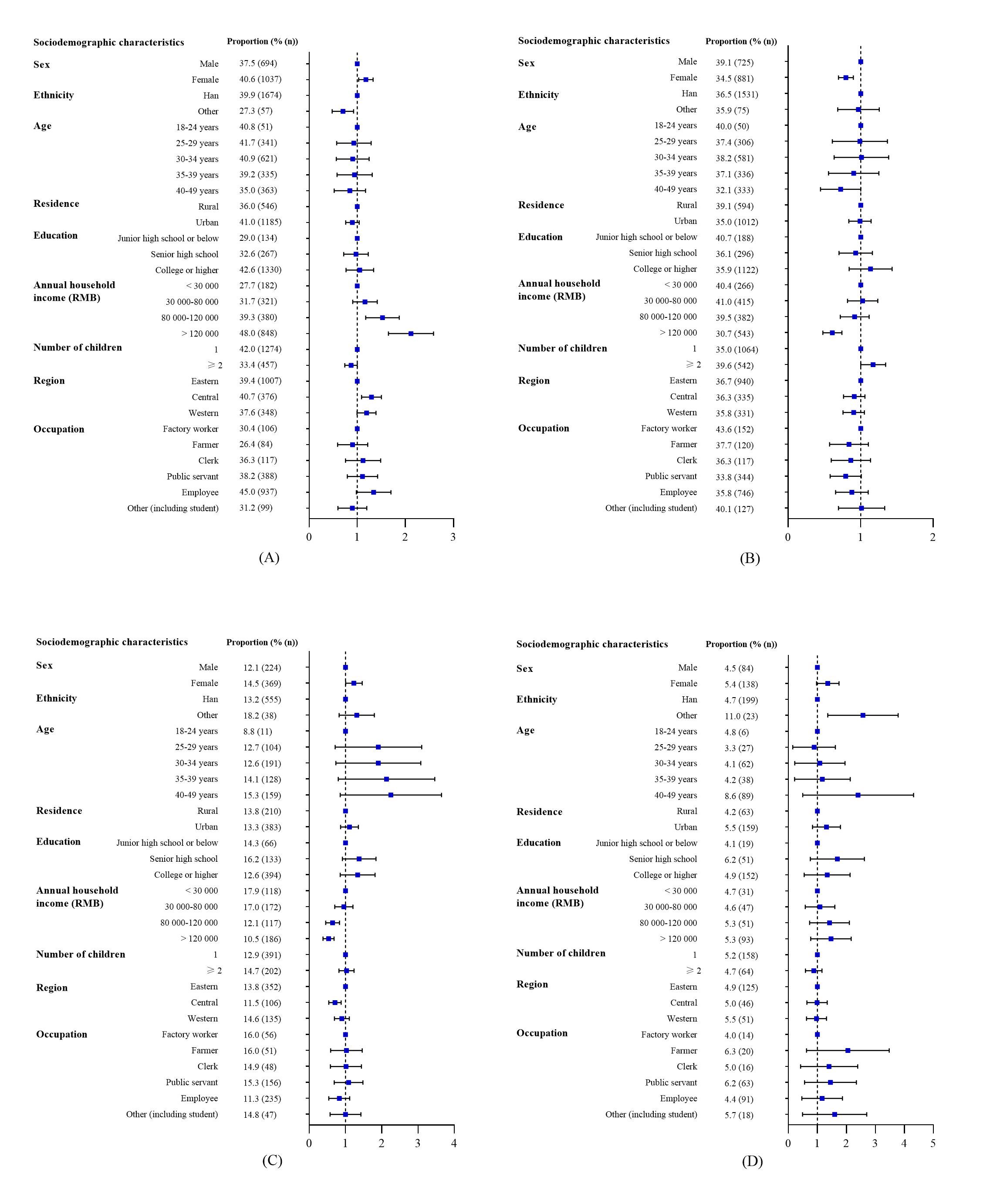

Supplement: Supplementary file 3 — Additional file 3: Supplemental Fig. 2. The biggest barrier to rearing children aged 0–3 years among our study population with different sociodemographic characteristics. (A) High time cost, (B) High childrearing cost, (C) High educational cost, (D) Physical factors. [file 12889_2022_12880_MOESM3_ESM.jpg]
